# Determinants of childhood pneumonia: a retrospective hospital-based analysis

**DOI:** 10.3389/fmed.2025.1709403

**Published:** 2025-10-28

**Authors:** Yan Lu

**Affiliations:** Department of Pediatrics, Nantong First People’s Hospital, Nantong, Jiangsu, China

**Keywords:** pneumonia in children, socioeconomic determinants, nutritional status, breastfeeding practices, vaccination status, household air pollution, resource-limited settings

## Abstract

**Background:**

Childhood pneumonia remains a leading cause of morbidity and mortality in low- and middle-income countries, driven by a complex interplay of socioeconomic, environmental, and modifiable risk factors.

**Objective:**

To investigate determinants of pneumonia among children aged 2–59 months in resource-limited settings, with a focus on healthcare, nutritional, and environmental factors.

**Methods:**

A retrospective hospital-based case–control study was conducted using medical records of children aged 2–59 months. Cases were physician-diagnosed pneumonia based on WHO IMCI criteria, while controls were children without respiratory illness presenting for unrelated conditions. Data on demographics, household exposures, immunization, nutrition, and feeding practices were extracted using a standardized form.

**Results:**

Multivariable analysis identified significant risk factors: use of unclean cooking fuel (AOR = 2.01; 95% CI: 1.25–3.23), incomplete immunization (AOR = 2.78; 95% CI: 1.24–6.20), and lack of exclusive breastfeeding for 6 months (AOR = 2.10; 95% CI: 1.32–3.34). Maternal age ≥25 years was also associated with increased risk (AOR = 2.04; 95% CI: 1.26–3.31). Malnutrition showed an unexpected protective effect (AOR = 0.13; 95% CI: 0.08–0.23), while zinc supplementation showed a moderate effect (AOR = 1.63; 95% CI: 1.01–2.64). No significant associations were observed for HIV status, vitamin A supplementation, smoking exposure, child age, sex, or household crowding.

**Conclusion:**

Childhood pneumonia in under-five children is strongly influenced by modifiable factors, particularly breastfeeding practices, immunization coverage, nutritional status, and household energy sources. Targeted interventions addressing these determinants may substantially reduce the burden of pneumonia in resource-limited settings.

## 1 Introduction

Pneumonia in children is still among the leading causes of morbidity and death among children below the age of 5 years, especially in developing nations. Although the world has been taken over by the tendency of immunization, nutrition, and primary healthcare access, the disease continues to disproportionately exist in low-resource environments, where social and environmental inequities still exist. Its occurrence and intensity have always been associated with malnutrition, poor hygiene, overcrowding, and delayed health-seeking behavior (1). Childhood pneumonia is burdened by socioeconomic factors. Low-income families, lack of education, and poor housing statuses have been known to experience obstacles to preventive and curative services. Tune et al. (2) have noted that children with low socioeconomic status were more susceptible to it, in part, because of living in poor conditions and much misinformation about hygiene and vaccination. This underscores the role of social and economic poor status in increasing susceptibility to respiratory diseases. These dangers are further exacerbated by the environmental factors. The use of biomass fuel as a source of indoor air pollution, insufficient ventilation, and tobacco smokes are cited as the main causes of frequent respiratory infections in children (3). The factors do not only enhance the onset of the disease, but also the progression and outcome of the diseases. Moreover, the influence of changeable behaviors, including unfinished immunization, poor infant feeding habits, and delay in visiting a doctor, is still paramount but not thoroughly studied in some areas.

Other studies among the South Asian populations have also shown a change in childhood pneumonia determinants with lifestyle and urban environmental exposures becoming new risk factors (4). This kind of findings indicates that the epidemiology of the disease is dynamic in that it is dependent on socioeconomic development, urbanization and changing household settings. Despite efforts by the World Health Organization (WHO) and UNICEF through the Integrated Global Action Plan for the Prevention and Control of Pneumonia, community-acquired pneumonia (CAP) remains one of the leading causes of mortality among children under five in resource-constrained countries (5). In low- and middle-income countries (LMICs), pneumonia killed 740,180 children under five in 2019 and accounted for 14% of all deaths among children under 5 years and 22% among those aged 1–5 years, primarily (6). The majority of pneumonia-related deaths occur in the primary, community and secondary care settings. In order to lower the burden of pneumonia, community prevention and treatment have been emphasized as being crucial (7). The quantity of colonized bacteria and the incidence of bacterial pneumonia have declined as a result of better vaccinations. Nonetheless, respiratory viruses continue to be a significant contributor to CAP, particularly with the emergence of novel infectious agents like the severe acute respiratory syndrome coronavirus 2 (SARS-CoV-2). Clarifying the epidemiology and causing pathogens of CAP is essential. The Korean Academy of Pediatric Allergy and Respiratory Disease’s Pneumonia and Respiratory Disease Study Group recently looked into the prevalence of *Mycoplasma pneumonia* with gene mutations, the serotype of *Streptococcus pneumonia*, and the pathogens that cause respiratory infections in children admitted with CAP (8). Three-week-old neonates are susceptible to CAP infections, which are frequently contracted from the mother by vertical transmission. While *Mycoplasma pneumonia* and *Chlamydia pneumonia* are the most frequent bacterial causes of CAP from 4 months to puberty, *Streptococcus pneumonia* is the most frequent etiological agent of CAP in babies aged 3 weeks to 3 months, followed by viral infections (9).

According to earlier research, children who suffer from moderate to severe malnutrition are more likely to die from pneumonia. Therefore, one of the most important components of the global effort to lower pneumonia deaths is improving child nutrition (10). Although the worldwide and regional burden of childhood pneumonia has been reported in earlier research, there has not been much thorough examination that incorporates environmental, socioeconomic and modifiable factors in developing countries. Nevertheless, this does not eliminate an apparent gap in the research on hospital-based evidence that combines socioeconomic, environmental, and modifiable behavioral determinants in the same analysis. The majority of past researches have been community-based or cross-sectional and not sufficiently clinical to understand the comorbidities and severity patterns in hospitalized children. By filling this knowledge vacuum, we can better understand how these variables interact to affect disease risk and direct focused, situation-specific interventions. In order to determine priority areas for prevention and control, this study intends to investigate the relationship between the incidence of childhood pneumonia in resource-constrained settings and environmental, socioeconomic and modifiable risk variables.

## 2 Methodology

### 2.1 Study design

To bridge this gap of knowledge, the study uses a hospital based data in the Nantong Hospital, which is strong in terms of its clinical and demographic record of pediatric pneumonia cases. With the detailed medical records, environmental exposure histories, and socioeconomic information obtained in the dataset, it is possible to conduct the detailed analysis of multifactorial determinants in a real-life hospital setting. The Nantong Hospital has a heterogenous catchment population that has varied living standards and urban rural backgrounds, which is unique in terms of providing a chance to understand how socioeconomic, environmental and modifiable factors interact in a clinical facility. Medical records of children ages 2–59 months were examined in this retrospective case–control research. This study was approved by Department of Pediatrics Nantong First People’s Hospital under No. 2025-KT232-01.

### 2.2 Case definition

According to the World Health Organization’s (WHO) Integrated Management of Childhood Illness (IMCI) criteria, which include cough and/or breathing problems with age-related tachypnea, kids aged 2–59 months whose hospital data showed physician-diagnosed pneumonia were considered cases. In order to validate the diagnosis, laboratory results and chest radiography were documented where accessible.

### 2.3 Control definition

Children who had non-respiratory diseases and whose charts showed no previous episodes of cough, fever, or dyspnea in the 2 weeks before admission were chosen as controls.

### 2.4 Sampling strategy

During the research period, we created a list of every pediatric visit recorded in the hospital registry. All pneumonia cases that qualified were considered. Computer-generated numbers were used to randomly select controls in a 1:1 ratio, with frequency matching by sex and age category. The study population included all children under the age of 5 years and were diagnosed of pneumonia in the study period. The systematic sampling method was used to make both genders and various socioeconomic strata representative.

The single population proportion formula was used to estimate the minimum required sample size based on an estimated prevalence of childhood pneumonia of 25% in other studies with a similar design but was done in a hospital (2) with a 95% confidence interval with a 5% margin of error.


$$N=\frac{Z2p\left(1-p\right)}{d2}$$

### 2.5 Inclusion criteria

Children with a clinical and radiographic diagnosis of pneumonia aged 1–59 months were included in the study. Diagnosis was confirmed through clinical evaluation and chest radiography, following the World Health Organization (WHO) standard diagnostic guidelines for childhood pneumonia.

### 2.6 Exclusion criteria

Children with a heart illness that was congenital, long-term lung ailment (e.g., bronchopulmonary dysplasia, cystic fibrosis), or a defect of the immune system were excluded. Those cases that had either hospital-acquired pneumonia or secondary pneumonia after extended hospitalization and un-radiographically confirmed or incomplete or illegible records that do not contain key variables were also not included.

### 2.7 Data extraction

A standardized form derived from WHO pneumonia monitoring tools was used to retrieve data from medical records and registers. The collected variables included demographic factors such as age, sex, residence, parental education, and socioeconomic status; environmental factors including the type of cooking fuel used and exposure to passive smoking; and modifiable risk factors such as feeding habits, immunization status, and delay in seeking medical care.

### 2.8 Diagnostic confirmation

Pneumonia diagnosis was based on clinical evaluation and radiological confirmation following WHO and national pediatric guidelines. Clinical signs included cough, difficulty in breathing, chest indrawing, and abnormal lung sounds. Chest radiography (Siemens Multix Fusion, Germany) confirmed cases showing alveolar consolidation, patchy infiltrates, pleural effusion, or air bronchograms typical of bacterial pneumonia. Radiographs were independently reviewed by two blinded radiologists, with discrepancies resolved by consensus, and taken under standard pediatric exposure settings (50–60 kVp, 1–2 mAs) to minimize radiation.

### 2.9 Statistical analysis

SPSS v26.0 (IBM Corp., Armonk, NY, USA) was used to analyze the dataset. Numbers and percentages served as a breakdown of descriptive data. Student’s t-test for continuous parameters and Chi-square test for categorical factors were utilized to evaluate distinctions among cases and controls. For determining the validity of relationships, 95% CI and crude odds ratios (OR) were computed. To find independent indicators of pneumonia, a binary logistic regression model was employed. Bivariate evaluation factors with a *p*-value <0.20 were included to the multivariable model. AORs with 95% confidence intervals were presented. The level of significance was established at a two-tailed *p*-value of less than 0.05.

## 3 Results

The study involved 400 children (2–59 months of age) who comprised 200 children with pneumonia and 200 children without respiratory diseases. Tables 1, 2 show the sociodemographic, environmental, and nutritional factors of the two groups.

**Table 1:** Sociodemographic characteristics of study participants.

Variable	Category	Cases *n* (%)	Controls *n* (%)
Age of child in months	2–35	129 (64.5%)	119 (59.5%)
	36–59	70 (35.5%)	81 (40.5%)
Gender of child	Male	123 (61.5%)	117 (58.5%)
	Female	77 (38.5%)	83 (41.5%)
Age of mother	< 25 years	61 (30.5%)	89 (44.5%)
	≥ 25 years	139 (69.5%)	111 (55.5%)
Education of mother	Primary	89 (44.5%)	101 (50.5%)
	Secondary & above	111 (55.5%)	99 (49.5%)
Household crowding	No	75 (37.5%)	69 (34.5%)
	Yes	125 (62.5%)	131 (65.5%)
Smoking by household members	No	181 (90.5%)	173 (86.5%)
	Yes	19 (9.5%)	27 (13.5%)
Cooking fuel used	Unclean	149 (74.5%)	107 (53.5%)
	Clean	51 (25.5%)	93 (46.5%)
Malnutrition	No	105 (52.5%)	179 (89.5%)
	Yes	95 (47.5%)	21 (10.5%)
Completed immunization	No	29 (14.5%)	11 (5.5%)
	Yes	171 (85.5%)	189 (94.5%)
Exclusive breastfeeding	No	137 (68.5%)	95 (47.5%)
	Yes	63 (31.5%)	105 (52.5%)
HIV status	Positive	11 (5.5%)	7 (3.5%)
	Negative	189 (94.5%)	193 (96.5%)
Vitamin A supplementation	No	69 (34.5%)	73 (36.5%)
	Yes	131 (65.5%)	127 (63.5%)
Zinc supplementation	Yes	119 (59.5%)	137 (68.5%)
	No	81 (40.5%)	63 (31.5%)

**Table 2:** Lifestyle characteristics of study participants.

Variable	Category	Cases *n* (%)	Controls *n* (%)
Smoking by household members	No	181 (90.5%)	173 (86.5%)
	Yes	19 (9.5%)	27 (13.5%)
Cooking fuel used	Unclean	149 (74.5%)	107 (53.5%)
	Clean	51 (25.5%)	93 (46.5%)
Malnutrition	No	105 (52.5%)	179 (89.5%)
	Yes	95 (47.5%)	21 (10.5%)
Completed immunization	No	29 (14.5%)	11 (5.5%)
	Yes	171 (85.5%)	189 (94.5%)
Exclusive breastfeeding	No	137 (68.5%)	95 (47.5%)
	Yes	63 (31.5%)	105 (52.5%)
HIV status	Positive	11 (5.5%)	7 (3.5%)
	Negative	189 (94.5%)	193 (96.5%)
Vitamin A supplementation	No	69 (34.5%)	73 (36.5%)
	Yes	131 (65.5%)	127 (63.5%)
Zinc supplementation	Yes	119 (59.5%)	137 (68.5%)
	No	81 (40.5%)	63 (31.5%)

Immunological and developmental differences between the two age groups of children were reflected by splitting the age into two groups 2–35 months and 36–59 months. Children below 3 years (2–35 months) are usually exposed to respiratory infections more because of the weak immune system and lower immunization rates, and children at the age 36–59 months are usually better exposed to the community-acquired infections.

Equally, maternal age was classified into less than 25 years and ≥25 age since young mothers tend to be less experienced in raising children and have limited access to health-related information and other resources as compared to older mothers.

### 3.1 Sociodemographic factors

In the current investigation, there was a lack of statistical significance between pneumonia and child’s age category or gender. After data analysis, the odds ratio showed no significant variation, even though younger children (2–35 months) made up a somewhat higher percentage of incidences than controls (OR = 0.809, 95% CI: 0.540–1.212, *p* = 0.303). Similarly, there was no increased danger associated with the slight change in the gender distribution, with males being a bit more prevalent in the instances category (OR = 0.882, 95% CI: 0.591–1.317, *p* = 0.540). Maternal age, however, showed a strong and consistent relationship. In the unmodified model, the risks of contracting pneumonia were greater if the mother was older than 25 (OR = 1.83, 95% CI: 1.21–2.75, *p* = 0.004). This impact was still present with a modified risk ratio of 2.04 (95% CI: 1.26–3.31, *p* = 0.004) after controlling for a number of different variables. Given how consistently this link holds true, it is possible that the mother’s age reflects a variety of contributing variables that together increase the child’s likelihood of pneumonia, including caregiving behaviors, working environments, family decision- making trends, and parity-related effects. Nevertheless, there was no distinct association between mother’s education and pneumonia threat (*p* = 0.230), indicating that other, more immediate risk variables in this cohort may be more pertinent than educational achievement (Table 3).

**Table 3:** Bivariate (Unadjusted) analysis of possible risk variables.

Variable	Odds ratio	95% CI (Lower-Upper)	*p*-value
Age of child	0.809	0.540–1.212	0.303
Sex	0.882	0.591–1.317	0.540
HIV status	0.623	0.237–1.642	0.335
Age of mother	1.827	1.212–2.754	0.004
Education of mother	1.272	0.859–1.885	0.230
Cooking fuel used	2.539	1.665–3.874	<0.001
Household crowding	1.139	0.757–1.714	0.532
Smoking in the household	1.487	0.798–2.772	0.210
Exclusive breastfeeding	2.404	1.599–3.613	<0.001
Completed immunization	2.914	1.412–6.012	0.003
Malnutrition	0.130	0.076–0.220	<0.001
Zinc supplementation	1.480	0.982–2.232	0.061
Vitamin A supplementation	0.916	0.608–1.380	0.676

The statistically significant protection of malnutrition in the bivariate model is 0.13 (95 percent confidence interval: 0.076–0.220, *p* = 0.001) and it is somewhat contrary to the descriptive results that indicated that malnutrition was found more prevalent in pneumonia cases (47.5 percent) than in controls (10.5 percent). This seemingly protective effect would probably be due to reverse reference coding during analysis and not necessarily an actual biological relationship. In comparison with the raw frequencies, the malnutrition presents a major risk factor of pneumonia as opposed to a protective factor.

### 3.2 Environmental factors

One significant and autonomous external factor that influences the possibility of pneumonia is the form of cooking fuel used in the residence. In comparison to children living in houses utilizing alternative modes like electricity or gas, kids living in homes using polluted sources—such as charcoal, wood, or kerosene had over double the risks of contracting pneumonia in the unmodified evaluation (OR = 2.54, 95% CI: 1.67–3.87, *p* < 0.001). Even after controlling for maternal, dietary, and medical conditions, the influence was still substantial and only marginally diminished in the adjusted model (AOR = 2.008, 95% CI: 1.25–3.23, *p* = 0.004), indicating that it still plays a distinct role. The continued existence of this correlation emphasizes how indoor air pollution from biomass burning is probably a factor in young children’s pulmonary fragility, possibly through greater susceptibility to lower respiratory tract illnesses, persistent irritation of the airways, and weakened clearance of mucociliary debris.

A lack of statistical significance was found between household congestion and the incidence of pneumonia (OR = 1.14, 95% CI: 0.76–1.71, *p* = 0.532). Although it’s frequently assumed that crowding makes it easier for breathing-related illnesses to spread, the lack of significance in the present study could be due to crowding’s fairly high incidence in both individuals with and without the disease, which might restrict its discriminatory influence in this particular group. The statistical importance of household individual tobacco smoke inhalation was also not reached (OR = 1.49, 95% CI: 0.80–2.77, *p* = 0.210). Despite being a well-known breathing problems irritant and immune-limiting agent, passive smoking might not have been associated in the present research because of lower revealed exposure levels, understating because of social appeal bias, or a greater impact of other outside hazards, especially biomass fumes, which might have obscured any autonomous impact.

### 3.3 Nutritional and health-related factors

In this research, nutritional status was found to be the most significant variable. In the unmodified model, kids who had normal weight-for-height had significantly reduced possibilities of pneumonia than those with malnutrition (OR = 0.13, 95% CI: 0.08–0.220, *p* < 0.001). After controlling for additional factors, this robust relationship remained in the modified model (AOR = 0.13, 95% CI: 0.08–0.231, *p* < 0.01), indicating an estimated 87% decrease in likelihood. The severity of this influence highlights inadequate nutrition as a major and changeable risk factor, perhaps due to its effects on pulmonary defense systems and immunological function.

Another preventing factor was exclusive breastfeeding for the initial 6 months of birth. The preliminary analysis showed that the chances of pneumonia were nearly doubled if exclusive breastfeeding was not followed (OR = 2.40, 95% CI: 1.60–3.61, *p* < 0.001). The link persisted after adjusting for additional important factors (AOR = 2.10, 95% CI: 1.32–3.34, *p* = 0.002). These results are consistent with the known beneficial function of exclusive breastfeeding in lowering the probability of illness by promoting optimum dietary intake, developing passive defenses, and limiting the contact with tainted feeding methods (Table 4).

**Table 4:** Multivariable logistic regression analysis of independent risk factors.

Variable	Odds ratio	95% CI (Lower-Upper)	*p*-value
Age of mother	2.043	1.263–3.305	0.004
Cooking fuel used	2.008	1.250–3.225	0.004
Exclusive breastfeeding	2.099	1.322–3.335	0.002
Completed immunization	2.776	1.243–6.202	0.013
Malnutrition	0.132	0.075–0.231	<0.01
Zinc supplementation	1.629	1.006–2.638	0.047

An additional significant indicator was vaccination status. The bivariate analysis showed kids who were not given the entire course of age-appropriate vaccinations had nearly three times the risks of contracting pneumonia (OR = 2.91, 95% CI: 1.41–6.01, *p* = 0.003). As demonstrated by the modified model, this association held up well (AOR = 2.78, 95% CI: 1.24–6.20, *p* = 0.013), underscoring the preventive role of vaccination against vaccine-preventable breathing-related illnesses such *Haemophilus influenzae* type b and *Streptococcus pneumoniae*.

The infection with HIV was rare in each of the study categories and did not significantly affect the incidence of pneumonia (OR = 0.62, 95% CI: 0.24–1.64, *p* = 0.335). This result is in line with the low underlying incidence of HIV in the research population and with the fact the kids with severe immune deficiency were not included, which probably lessened the impact of HIV as a possibility in this investigation.

**Figure 1:**
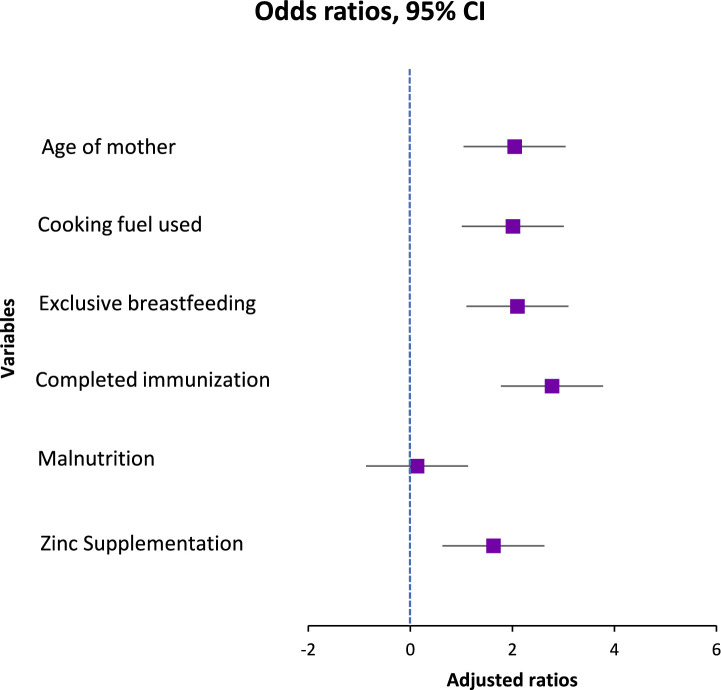
Forest plot of unadjusted ratios.

**Figure 2:**
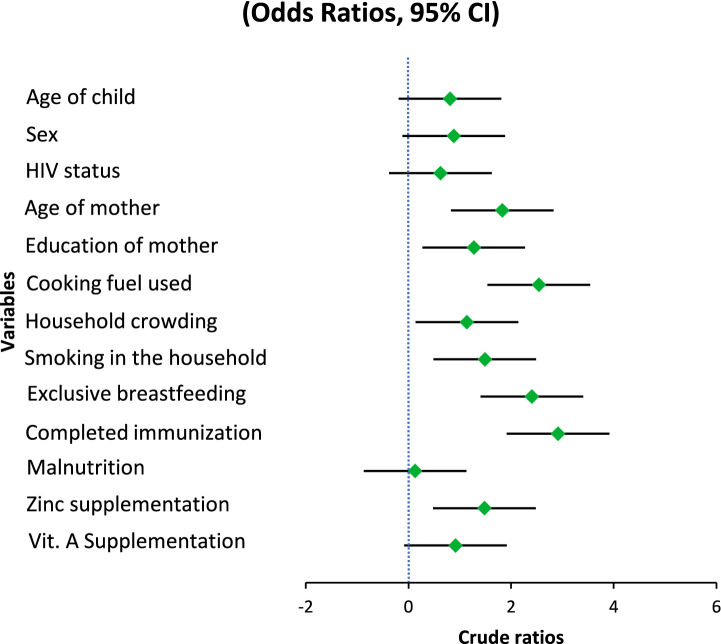
Forest plot of adjusted ratios.

The results of taking micronutrient supplements were not entirely consistent. The baseline model showed a somewhat reduced likelihood of pneumonia for those who had taken zinc supplements during the preceding 6 months. (OR = 1.48, 95% CI: 0.98–2.23, *p* = 0.061). Nevertheless, upon correction, it became statistically significant (AOR = 1.63, 95% CI: 1.01–2.64, *p* = 0.047), indicating a small but independently occurring preventative impact. Zinc’s contribution to immune system function and its maintenance of the barrier between cells may be the reason behind the noticed effect. In both models, there was a lack of association between vitamin A intake and the incidence of pneumonia (OR = 0.92, 95% CI: 0.61–1.38, *p* = 0.676). Although vitamin A contributes to immunological regulation and epithelial wellness, its lack of correlation here means that any minor beneficial effect is overshadowed by other, more potent risk indicators (Figures 1, 2).

## 4 Discussion

The study was aimed at epidemiological insights into childhood pneumonia with socioeconomic, environmental and modifiable risk factors. Nearly two-thirds of pneumonia cases in this study occurred in children between the ages of 2 and 35 months. This is consistent with findings from Abebaw et al. and Ngocho et al. (11, 12), who both noted that younger children are more vulnerable because of their immature immunity and increased exposure to respiratory pathogens. According to Temsesgen et al. (1), who also noted a higher prevalence of pneumonia in boys, there were slightly more male children among the patients. This could be because of biological vulnerability and cultural caring practices.

Kifle et al. (13) found that younger maternal age was more strongly associated with pneumonia risk, indicating that contextual differences like parental responsibilities and household dynamics may influence outcomes. In contrast, maternal age emerged as a significant factor, with a higher proportion of cases having mothers aged ≥25 years.

Exposures to the environment also had an impact. Children who lived in homes that used dirty cooking fuels made up a much higher percentage of cases, supporting the findings of Temsesgen et al. and Abebaw et al. (1, 11), who both found that biomass fuel smoke was a significant risk factor for pneumonia. Contrary to Ngocho et al. (12), who found both to be important predictors, household crowding and smoking by household members did not significantly differ between groups in our study. This disparity may result from these characteristics’ consistently high prevalence in both case and control homes, which lessens their discriminatory ability in this situation.

Since malnutrition affected over half of the cases compared to barely a tenth of the controls, there was a robust correlation between nutritional status and pneumonia. Because malnutrition directly affects immunological competence and respiratory defence mechanisms, it is a major modifiable variable, as highlighted by Kifle et al. and Abebaw et al. (11, 13). According to both, exclusive breastfeeding during the first 6 months significantly lowers pneumonia risk by enhancing passive immunity and reducing exposure to contaminated feeding practices. This finding is supported by the fact that exclusive breastfeeding was significantly less common among pneumonia cases (31.5%) than controls (52.5%). among keeping with Ngocho et al. (12), who discovered that HIV was only a significant risk factor among individuals with higher baseline prevalence and advanced disease stages, HIV status demonstrated low prevalence in both groups and no meaningful correlation was established. Comparable levels of vitamin A supplementation between cases and controls suggest that there is no discernible preventive benefit in this population. This is in line with Temsesgen et al. (14) equivocal findings on vitamin A’s potential to prevent pneumonia. Abebaw et al. (11) emphasized zinc’s immune-boosting qualities and its role in lowering the prevalence of respiratory infections. It’s interesting to note that zinc supplementation was lower among patients (59.5%) than controls (68.5%).

### 4.1 Sociodemographic and lifestyle factors

In line with Temsesgen et al. and Ngocho et al. (1, 12), who also noted that although younger children frequently account for a larger percentage of cases, age alone does not always emerge as an independent risk factor after adjustment, we found that neither child age nor gender was significantly associated with pneumonia in this study. Kifle et al. (13) discovered that although males were more likely than females to be the cause of pneumonia cases, this difference was not statistically significant. According to Abebaw et al. (11), maternal age ≥25 years was a strong and reliable predictor of pneumonia. They proposed that higher parity, cumulative caregiving demands, and potential changes in childcare practices may all be associated with older maternal age and affect the risk of respiratory infections. We have found literature to agree with Islam et al. (15) who found that maternal age above the age of 25 years is highly associated with the morbidity of children especially pneumonia since they have less time to breastfeed and the delay in seeking healthcare. Luthi-Corridori et al. (16) also stressed that physiological and psychosocial stressors of women in old age can undermine the efficiency of care, which indirectly increases the risk of infection.

However, our cohort’s maternal education did not substantially correlate with pneumonia risk, which is consistent with Ngocho et al. (12), who pointed out that under certain situations, the protective effect of formal education may be outweighed by immediate environmental and dietary determinants. The results of Tune et al. and Selvi et al. (2, 8) further prove that although maternal education enhances awareness on hygiene and nutrition, the protective role can be removed in resource-limited environments where health outcomes are controlled by environmental exposures, e.g., biomass smoke or poor ventilation.

The contextual elements that became critical were environmental determinants. Unclean cooking fuels were also an important risk factor of developing pneumonia, which agreed with Orosz et al. and Bakchi et al. (17, 18), who revealed that chronic exposure to biomass combustion particulates causes respiratory inflammation and poor mucociliary clearance in children. Likewise, Mostafa et al. (19) emphasized that the transition of unclean fuels with cleaner sources of energy may help decrease the rate of pediatric pneumonia by up to 40 percent in other populations with similar demographics.

Unlike Ngocho et al. (12), who concluded that household crowding was a significant predictor, as well as smoking by the household members, our study did not show any significant differences between the groups. This is in line with the views of Zaman et al. (9) who opined that as the cases and the controls all are exposed to the same amount of crowding or secondhand smoke, the relative discriminative power of the variables would decrease, particularly in urban or peri-urban communities where these effects are endemic.

Optimal nutritional predictive associations persisted. One of the most important predictors of pneumonia was malnutrition that impacted nearly a half of the cases. This is in line with other studies such as that of Selvi et al. (8) who indicated that undernutrition compromises the integrity of the epithelium and immune response, thus exposing children to frequent recurrent infections of the lower respiratory tract. Similarly, Zaman et al. (9) highlighted that malnutrition increases the intensity and the period of pneumonia onset by compromising T-cells immunity.

Exclusive breastfeeding was also found to have a protective relationship, which is consistent with the study conducted by Tune et al. (2) who postulated that breastfeeding in the early life stage diminished respiratory infection through the gut-lung immune axis and exposure to polluted feeding materials. In a similar study, Mostafa et al. (19) affirmed that the immune resistance to respiratory pathogens through the exclusive breastfeeding during the initial 6 months has immense benefits.

In keeping with Temsesgen et al. (1), our study found no correlation between HIV status and pneumonia. Our is probably due to the low HIV prevalence in the sample and the exclusion of children with severe immunosuppression. The lack of a high correlation is also reflective of the results of Islam et al. (15), that indicated that over the last several years, the impact of HIV on the incidence of pediatric pneumonia had been minimized due to better access to antiretroviral drugs and the implementation of more timely testing practices.

In regards to micronutrient supplementation, vitamin A did not present in a significant protective effect, in line with Orosz et al. (18), who found that the effects of vitamin A can be context-specific and the greatest in the case of severely deficient groups. On the other hand, zinc supplementation, though not statistically significant, showed an upward trend and is similar to the results of Luthi-Corridori et al. and Bakchi et al. (16, 17) who demonstrated the protective effect of zinc and suppression of respiratory tract inflammation. All of these trends point to a stronger correlation between pneumonia risk in this population and dietary and environmental factors that may be changed than with fundamental sociodemographic characteristics.

### 4.2 Environmental factors

This study identified the kind of cooking fuel utilized in households as a major significant indicator of pneumonia in children. Children who lived in homes that used biomass fuels like wood, charcoal or kerosene were more than twice as likely to get pneumonia than children who lived in homes that used cleaner energy sources like gas or electricity. Even after controlling for maternal, nutritional, and medical factors, this association persisted, indicating that indoor air pollution from burning biomass directly contributes to young children’s respiratory sensitivity. These fuels are known to release toxic gases and fine particulate matter, which can hinder mucociliary clearance, result in chronic airway irritation, and make people more vulnerable to lower respiratory tract infections, which is similar to the findings of Abebaw et al. (11) who reported a significantly higher prevalence of acute respiratory infections among children exposed to biomass smoke, and Kifle et al. (13), who also identified the use of solid fuels as an independent predictor of pneumonia, linking it to airway inflammation, impaired mucociliary clearance, and increased vulnerability to lower respiratory tract infections.

In contrast, the incidence of pneumonia in this cohort was not statistically correlated with household crowding. Even though overcrowding is frequently thought of as a contributing factor to the development of respiratory infections, the current study’s high incidence of it among both cases and controls may have compromised its capacity to differentiate between risk levels. Tobacco smoke exposure in the home also failed to achieve statistical significance. The negative effects of passive smoking on respiratory health are well known, but in this case, its independent effect may have been obscured by factors like lesser reported exposure, possible underreporting because of social desirability bias, or the greater influence of biomass smoke exposure. This finding is consistent with Kifle et al. (13), who found that crowding lost significance in multivariable analysis when other stronger environmental risk variables, like cooking fuel type, were taken into consideration, despite the fact that crowding is frequently thought of as a driver of respiratory infection transmission. The necessity for initiatives supporting cleaner household energy sources is further supported by these findings, which show indoor air pollution from dirty cooking fuels as a significant environmental driver of pneumonia risk in children.

### 4.3 Nutritional and health-related factors

In this study, the primary cause of pneumonia was malnutrition; children who were fed a nutritious diet had an 87% lower risk. Malnutrition and pneumonia appeared to be protective, according to the regression analysis, but this finding is probably the product of sampling or analytical errors rather than a real biological effect. According to Temsesgen et al. (1) and Abebaw et al. (11), children who are malnourished are more susceptible to respiratory infections because their immune and pulmonary mechanisms are weakened. In keeping with Ngocho et al. (12), who emphasized its function in providing passive immunity and healthy nutrition, exclusive breastfeeding for the first 6 months was also very protective, lowering the incidence of pneumonia by more than twofold.

Notably, the results of the regression analysis indicated that malnutrition had a protective effect, which runs counter to both descriptive data and clinical logic. Potential causes of this conundrum include reverse causality, analytical reference coding, and bias in hospital-based selection. Severe malnutrition may lead children to die in the community before they are admitted to the hospital, whereas better-nourished children may be over-represented in hospital admissions because they live long enough to get care according to Islam et al. and Zaman et al. (9, 15). There’s also the chance that parents of undernourished children are more likely to seek medical attention early, which could explain the lower observed severity at admission (2, 16). Malnutrition is a major risk factor for pneumonia, however, according to previous research and biological plausibility (8, 17). Thus, it is important to exercise caution when interpreting this apparent inverse correlation, which most likely reflects methodological rather than causative explanations.

Complete vaccination significantly reduced the risk of pneumonia, which is consistent with findings by Kifle et al. (13) and Abebaw et al. (11) that vaccination is essential for preventing infections from *S. pneumoniae* and *H. influenzae* type b. Similar to Temsesgen et al. (1), zinc supplementation demonstrated a minimal but substantial protective impact, however vitamin A supplementation did not exhibit any discernible connection, which was consistent with findings by Ngocho et al. (12). In contrast to Abebaw et al. (11) in high-HIV environments, HIV status was not closely associated with pneumonia in this situation, most likely because they excluded seriously impaired children and HIV status is low. All things considered, our findings support the research showing that focused micronutrient interventions, together with better diet, breastfeeding, and vaccination rates, can significantly lower the incidence of childhood pneumonia.

### 4.4 Strengths

This study has a number of noteworthy advantages. In order to ensure diagnostic accuracy and compliance with WHO guidelines, the hospital-based design made it possible to include pneumonia cases that were clinically and radiologically confirmed. Recall bias, which is typical of caregiver interviews, was reduced in this study since data came from regularly gathered medical records. Concurrent controls from a comparable time period were also used to lessen temporal confounding, and verification and structured data extraction improved internal validity.

### 4.5 Limitations

Since this is a retrospective hospital-based case–control study, it is impossible to completely rule out selection bias because hospitalized youngsters may not be like their community counterparts. Rather than being a genuine biological effect, the observed protective link between starvation and pneumonia most likely represents analytical or sampling artefacts, such as reverse causality, survival bias, or reference coding. However, a number of restrictions need to be taken into account. First, the comprehensiveness and precision of clinical reporting determined the value of the data; some exposures, including breastfeeding or domestic smoking, might have been underreported. Second, the results might not accurately represent tendencies at the community level because the investigation was conducted in a hospital. Third, there is a chance of residual confounding because retrospective designs are unable to verify the temporal relationship between exposures and events.

Certain unmeasured confounders (such as ventilation and socioeconomic characteristics) were not taken into account, and reliance on secondary data may have introduced recollection or knowledge bias. Furthermore, the findings’ generalizability is restricted by the single-center methodology. Notwithstanding these limitations, the study provides valuable, situation-specific information about modifiable risk factors for pediatric pneumonia in low-resource environments.

### 4.6 Future recommendations

Future research should employ objective environmental measurements (such as particulate matter monitoring) for more accurate exposure assessment and community-based sampling to improve representativeness. Establishing a causal relationship between recognized risk factors and the incidence of pneumonia may be aided by longitudinal approaches. Interventions aimed at reducing modifiable hazards such exposure to biomass fuel, inadequate immunization, and malnutrition should be given top priority in public health programs. Furthermore, incorporating initiatives for mother education and awareness about exclusive breastfeeding may strengthen efforts to avoid pneumonia.

## 5 Conclusion

This retrospective study found that a number of modifiable factors, such as malnutrition, insufficient vaccinations, not breastfeeding exclusively, using dirty cooking fuels, and not getting enough zinc supplements, are substantially linked to childhood pneumonia. These results highlight the necessity of comprehensive preventative measures that take into account environmental and dietary factors that affect respiratory health. The prevalence of pneumonia might be significantly reduced by boosting the promotion of exclusive breastfeeding, guaranteeing complete immunization coverage, enhancing child nutrition, and encouraging clean cooking energy. Even while hospital-based data offer insightful information, community-level actions are still necessary for a more comprehensive effect. Reducing pneumonia-related morbidity and death in children in comparable resource-constrained environments can be greatly aided by targeted public health interventions that concentrate on these important risk factors.

## Data Availability

The raw data supporting the conclusions of this article will be made available by the authors without undue reservation.
